# Capturing the Heterogeneity of Word Learners by Analyzing Persons

**DOI:** 10.3390/bs14080708

**Published:** 2024-08-13

**Authors:** Ian T. Jones, Sarah C. Kucker, Lynn K. Perry, James W. Grice

**Affiliations:** 1 Department of Psychology, Oklahoma State University, Stillwater, OK 74078, USA; ian.t.jones@okstate.edu; 2 Department of Psychology, Southern Methodist University, Dallas, TX 75205, USA; skucker@mail.smu.edu; 3 Department of Psychology, University of Miami, Coral Gables, FL 33124, USA; lkp36@miami.edu

**Keywords:** word learning, language development, person-centered, observation oriented modeling

## Abstract

Accurately capturing children’s word learning abilities is critical for advancing our understanding of language development. Researchers have demonstrated that utilizing more complex statistical methods, such as mixed-effects regression and hierarchical linear modeling, can lead to a more complete understanding of the variability observed within children’s word learning abilities. In the current paper, we demonstrate how a person-centered approach to data analysis can provide additional insights into the heterogeneity of word learning ability among children while also aiding researchers’ efforts to draw individual-level conclusions. Using previously published data with 32 typically developing and 32 late-talking infants who completed a novel noun generalization (NNG) task to assess word learning biases (i.e., shape and material biases), we compare this person-centered method to three traditional statistical approaches: (1) a *t*-test against chance, (2) an analysis of variance (ANOVA), and (3) a mixed-effects regression. With each comparison, we present a novel question raised by the person-centered approach and show how results from the corresponding analyses can lead to greater nuance in our understanding of children’s word learning capabilities. Person-centered methods, then, are shown to be valuable tools that should be added to the growing body of sophisticated statistical procedures used by modern researchers.

## 1. Introduction

Within the fields of developmental psychology and language development specifically, hypotheses are often posited for group-level phenomena. For example, researchers might ask whether children with language delays, as a group, score lower on an assessment than children without language delays, or whether children who receive a given intervention have a mean vocabulary size that differs from children who did not receive the intervention. However, as has become clear more recently, a subset of our hypotheses regarding developmental and language outcomes is actually questions about specific children [1,2,3,4,5], such as how an intervention may benefit an individual child. The goal with these hypotheses, then, is to conduct and disseminate research in a way that assists in understanding the development of the individual child. For language development researchers, this goal is especially important because if we only posit and test group-level comparisons, then the differences between specific individual children may go unexplained (e.g., [6]). For instance, Suzy, a child who has been identified as a late talker, may show improvements over the year in her expressive vocabulary development approaching her typically developing peers, but Charlie, also a late talker, may continue to lag behind. A group level focus—late talkers versus typical talkers—leaves questions as to why these two individuals differ unanswered (e.g., see [6,7,8]).

This is not to say that language development researchers have avoided alternative approaches to simple group mean comparisons. In fact, many language acquisition and word learning researchers have demonstrated that utilizing more complex statistics, such as mixed-effects regression, are beneficial in capturing variation between individual children’s word learning abilities [4,9,10,11,12,13]. Indeed, several articles and special issues throughout the field of infant development have emphasized the importance of providing more nuanced understandings of infant data (e.g., [4,11]), increasing the reliability of infant research (e.g., [14,15]), and improving best practices in infant research to create a more robust field (e.g., [16,17]). One particular effort to improve the field has focused on alternative statistical methods (e.g., Bayesian inference, mixed-effects models) that better capture variability across children (e.g., [4,11]). These alternatives have been shown to be superior in accounting for the heterogeneity in behavior across individual children compared to simple group mean comparisons. However, the findings from these more sophisticated analyses nevertheless are often reported as group-aggregated statistics such as group means, variances, standardized regression weights, and squared multiple correlations (i.e., *R*^2^). Individual children within a group, like possible Suzies and Charlies, can unfortunately still get lost in such statistics. As recently noted by van der Gaag [8], more person-centered approaches toward data analysis can help keep this from happening within the developmental sciences. 

Beyond dynamic structural equation modeling, Bayesian hierarchical modeling, latent pattern analysis, and hierarchical linear modeling, an alternative set of person-centered methods have recently been developed and tested. These methods are akin to traditional non-parametric statistics and typically revolve around a visual presentation and exploration of the data as well as the computation and interpretation of effect sizes based on raw, individual-level data (e.g., [7,18,19]). Arocha [20], Beechey [21], Erisman and Blom [22], de Klerk and colleagues [23], Sayette and colleagues [24], Speelman and McGann [19], Valentine and Buchanan [25], and Valentine and colleagues [26] have demonstrated the utility and effectiveness of these methods which offer two core advantages to the field of language development.

First and foremost, results revolve around a person-centered effect size which indicates the number of individuals in a study who behaved or performed according to theoretical expectation. Speelman and McGann [19] refer to this statistic as a “pervasiveness index” whereas Grice et al. [18] refer to it as the Percent Correct Classifications (PCC) index, and it is an effect size metric that can readily convey the “theoretical, practical, or clinical importance of results from various study designs and types of data” (Grice et al. [18], p. 9). Notably, developmental researchers have begun to advocate for an increased reliance upon and interpretation of effect sizes as opposed to solely relying on *p*-values (see [16,27,28]). The PCC index can clearly facilitate these efforts as it can be easily understood by trained scientists, professionals, or laypersons. When interpreting results from a study, a simple percentage can convey practical, clinical, and theoretical importance in a way not typically found amongst traditional effect sizes, such as Cohen’s *d*, *r*, or *η*^2^ (see also [29,30]). Furthermore, as Rutledge and Loh [31] state, “transforming [traditional] effect size values into real-world impact is often not a simple task” (p. 138). The PCC, however, is easily understood and can more readily be interpreted in terms of a study’s real-world impact. For example, there would be no ambiguity in interpreting the results of a study for which 80% of late-talking children improved following one therapeutic intervention compared to 50% of late-talking children who improved following a competing intervention. 

Second, analyses conducted with these person-centered methods do not require many of the numerous technical assumptions underlying both traditional and newer statistical methods, such as the continuous quantity assumption, the normality assumption, or random sampling assumption [32,33,34,35]. Of particular importance, even the more recently popular statistical methods, such as Bayesian inference and mixed-effects regression, require the assumption of random sampling [33,34,35], though this feat is rarely achieved in developmental work (e.g., [36,37]). By removing concerns about statistical assumptions made in the process of estimating population parameters, person-centered analyses encourage researchers to connect with the individuals in their studies by way of carefully constructed causal theories (see [18]). 

### Applications of Person-Centered Analyses to the Study of Language Development

The benefit of employing these person-centered methods can be demonstrated by exploring individual differences in late-talking and typically developing children. Understanding why some late-talking (LT) children catch up to their typically developing (TD) peers while others lag behind is critically important for language development researchers because of the consequences faced by LT children [38]. The LT children who do not catch up to their TD peers have an elevated likelihood of continued language delays, diagnosed disorders, and problems in academic, social, and general work settings [38,39]. In fact, some LT children will go on to develop developmental language disorder, putting them at further risk of delays [40,41,42]. Nonetheless, the majority of LT children will be within the normal range of language skills by grade school [38,43,44,45,46,47,48,49,50,51]. It is hence critical for language development researchers to identify factors that could explain this heterogeneity among LT children (see also [5,52,53]). One such area of recent work is LT children’s shape bias, or attention to shape features during word learning [4,54,55,56,57,58].

In the current study, we use data originally reported by Perry and Kucker from their 2019 study of novel noun generalization (NNG) and vocabulary knowledge. Perry and Kucker compared children’s choices in an NNG task in which an experimenter named a novel exemplar object and asked children to choose an item with the same name as the exemplar: a novel object matching the exemplar in shape, but not color or material (i.e., “shape match”), or a novel object matching the exemplar in material, but not color or shape (i.e., “material match”). Across trials, selecting shape matches more often than material matches is known as a “shape bias” (see [59]).

As a group, late-talking children tended to select the shape-matched item less frequently than typically developing children [4,56,57]. The development of the shape bias has previously been demonstrated to be associated with accelerations in vocabulary development [2,60,61,62]. Differences between children’s development of the shape bias have been tied to disparities in their vocabulary structure (e.g., [55,63]), such that children who know more object nouns naming categories organized by similarity in shape (e.g., “shape-based nouns”) are more likely to show a shape bias than those who knew fewer shape-based nouns, even after controlling for overall vocabulary size. Furthermore, children’s long-term language outcomes, such as whether or not they are diagnosed with developmental language disorder, are associated with both their tendency of showing a shape bias [54] and the proportion of shape-based nouns in their vocabulary as toddlers [5]. Indeed, in Perry and Kucker’s [4] work, they found that there was an association between the number of shape-based nouns in children’s vocabularies and the proportion of NNG trials on which they generalized the novel names by similarity in shape. However, the strength of this association was greater for typically developing children than late-talking children, highlighting the large amount of heterogeneity in the late talker population.

By comparing mixed-effects regression analyses to traditional group mean comparison methods (viz., *t*-tests and ANOVA), Perry and Kucker [4] showed how researchers can begin to account for the heterogeneity among samples in LT and TD children. In the analyses to follow we show how person-centered analyses can also be used to address heterogeneity in Perry and Kucker’s [4] sample of children while also providing answers to novel questions not raised by other traditional methods. In particular, we will show how a person-centered analysis allows researchers to ask novel questions and to focus upon specific late-talking children to prevent such cases from being lost in an aggregate form of analysis while simultaneously offering the tools necessary for explaining their differences in behavior.

## 2. Methods

### 2.1. Participants

The same sample of 64 children (*N*_Female_ = 26) from Perry and Kucker [4] were re-analyzed here. All children were between 16–30 months (*M* = 22 months) of age and monolingual English-speakers. Half were classified as late talkers (LTs) with productive vocabularies below the 30th percentile and were matched to a typically developing (TD) child on age and sex (see Perry and Kucker [4], p. 556). 

### 2.2. Procedures

**Figure 1:**
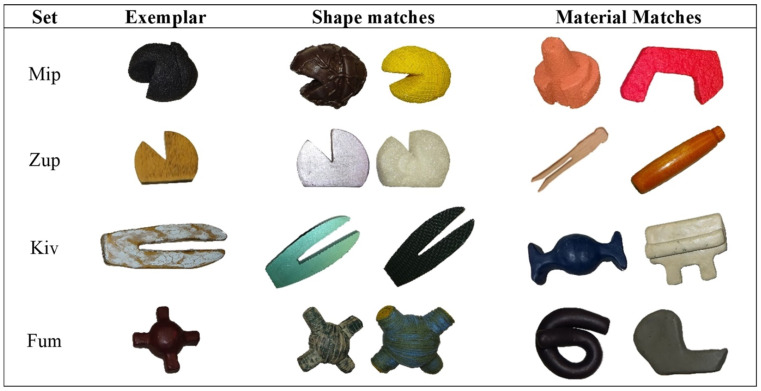
Novel noun generalization exemplars.

As described by Perry and Kucker [4], children completed a novel noun generalization (NNG) task to measure their tendency to select the shape-matched items. During the task, children were presented with a novel exemplar and an item that matched the exemplar in shape only and one that matched the exemplar in material only. The exemplar was named by the experimenter (e.g., “This is my kiv!”), and then the children were presented with the shape- and material-matching items and asked to select an item by name (e.g., “Can you get your kiv?”). Children’s choice of shape was coded offline by researchers blind to hypothesis and child language status. Four sets of items paired with names relatively equal in novelty and phonological density (viz., Kiv, Mip, Fum, and Zup; see Figure 1) were used, with four trials per set, for a total of 16 possible trials. Trials in which the child did not respond were removed and treated like missing data (including the analyses found in the Supplementary Files); thus, some children may have fewer than 16 total trials.

The TD and LT children had nearly equivalent rates of missing data (which typically occurred when a child refused to give a response), although the TD children had slightly more total missing cases and slightly more children missing at least one trial. As a group, the TD children successfully completed 467 out of 512 possible trials, with 45 total missing trials. In total, 12 out of 32 TD children (37.50%) had at least one missing trial. By comparison, the LT children successfully completed 458 out of 512 possible trials, with 54 total missing trials, and a total of 15 out of the 32 LT children (46.88%) had at least one missing trial (see Perry and Kucker [4], for further details).

### 2.3. Analytical Plan

In the analyses to follow, we demonstrate how one might conduct a *t*-test against chance, analysis of variance, and a mixed-effects regression with a person-centered perspective (the Observation Oriented Modeling software [64]), which can be freely downloaded, was used to conduct the analyses herein). Specifically, we conducted 3 pattern-based analyses (plus those found in the Supplementary Files) to analyze the quantity of shape choices for individual TD and individual LT children. In our case, these analyses will be utilized to analyze the frequency of shape choices across all 16 trials for TD and LT children. The analyses are presented in a sequential fashion such that we begin by comparing the concatenated pattern analysis against the *t*-test against chance, followed by the ANOVA, and finally the mixed-effects regression, including random effects. Finally, we included exploratory concatenated pattern analyses within the Supplementary Files to assess trial order effects.

## 3. Results

### 3.1. Person-Centered Tests against Chance versus Aggregate Tests of Chance

Do both TD and LT children select the shape-matching item, *on average*, significantly better than chance in the NNG task? With 0.50 as the expected mean value for chance, Perry and Kucker [4] performed single-sample *t*-tests to answer this question and concluded that, given the statistically significant results, “both sets of children performed better than chance” (p. 560). From a person-centered perspective, an additional question to be asked is “*which individual TD and LT children performed better than chance in the NNG task?*” Two advantages to asking this question are that (1) it permits us to go beyond average effects and focus on individual children, and (2) it aids in the exploration of potential heterogeneity of responses within the two groups.

**Figure 2:**
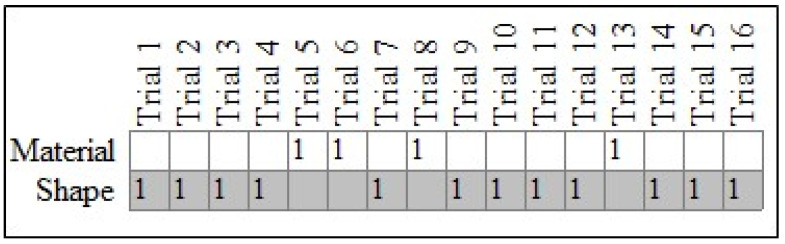
Responses for typically developing case #18. Note: Expected (gray cells) and observed (cells marked with a 1) patterns for case #18, a TD child.

To do this, a *pattern analysis*, which is based on the pattern of the individual responses of each child, was conducted. Figure 2 shows responses for an example TD child (case #18). Here, the figure is comprised of two rows with the top row representing the material choices and the bottom row showing shape choices. Each of the sixteen columns represent the trials, with 1s representing the child’s actual choice responses. The shaded cells for the “Shape” row represent our expectation that each child will exhibit a perfect tendency to select the shape-matched item.

For the particular child in Figure 2, 12 of their 16 responses were in the “Shape” row. Converting this result to a percentage yields a Percent Correct Classifications index (PCC) equal to 75.00%. The PCC index is essentially a person-centered effect size [18]) and it appears to be high for this child if 50% is adopted as a baseline for no preference to either material or shape. In addition to this index, the *pattern analysis* performs a randomization test to compute a distribution-free plausibility value, referred to as a chance-value (*c*-value; see [65,66]). (The randomization test is considered distribution free; however, in cases where the data are binary, like the current paper, the distribution will approximate the binomial distribution. In a more complex study design that includes multiple variables and varying numbers of categories, however, the sampling distribution may not be known. The randomization test can still be used in such complex study designs, thus providing researchers with a general tool for drawing inferences from their observations. Equally important, the randomization test serves as a reminder that the goal of the analysis is to draw an explanatory inference rather than an inference to a population parameter as is common with null hypothesis significance testing (see [66]). 

This value informs us as to whether the observed PCC could be explained by physical chance; in other words, it informs us whether these data could plausibly have been generated by accident. For example, could the responses for case #18 presented in Figure 2 above be randomly generated and an equivalent PCC or higher PCC be obtained? If the answer is yes, then we would conclude that the data can be best understood as a product of physical chance, or in simpler terms, as unsystematically or haphazardly produced. If the answer is no, then we would conclude that the pattern of responses is plausibly due to theoretically posited causal forces (viz., a shape bias is operating).

To compute the *c*-value for this design, we randomly determined the child’s observation of material or shape choice for each completed trial, re-computed the PCC from the pattern analysis, tallied whether the randomly generated PCC was greater than or equal to the observed PCC, and then repeated this process a set number of times (e.g., 10,000 iterations). The *c*-value was then computed by dividing the total sum of instances for which the randomized PCC was greater than or equal to the observed PCC by the total number of iterations. Mathematically, the process is represented as follows: $\frac{\sum_{i=1}^{k}{(PCC}_{k}\;\geq \;{PCC}_{obs})}{k}$; where *k* = the total number of iterations, PCC_k_ = the randomly generated PCC from the kth iteration, and PCC_obs_ = the observed PCC computed from the raw data. In the current paper, 10,000 iterations were utilized for all *c*-value computations. 

**Figure 3:**
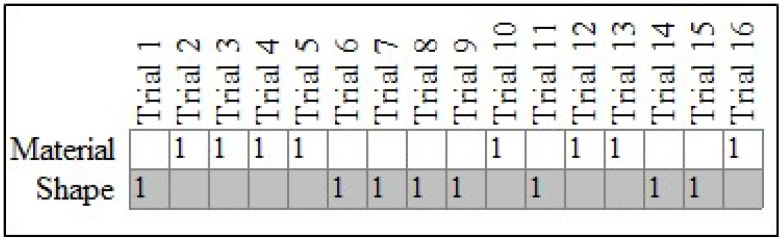
Responses for typically developing case #25. Note: Expected (gray cells) and observed (cells marked with a 1) patterns for case #25, a TD child.

For case #18 in Figure 2, the *c*-value from 10,000 iterations was equal to 0.04, thus indicating that their pattern of responses was not plausibly the result of physical chance. In other words, the child chose shape-matched items at a rate that exceeded what one would expect under conditions of physical chance. By way of comparison, Figure 3 shows the results for case #25, also a TD child. The PCC for their pattern was low (50.00%), and the *c*-value was high (0.60), thus indicating that their responses did not demonstrate a specific preference for either shape or material.

In Table 1, the left-hand columns report results for each of the 32 TD children, the overwhelming majority of whom had computed PCCs greater than 50% and low *c*-values (viz., <0.20), thus indicating a stronger preference to the shape-matched item. The PCCs varied somewhat (*absolute median deviation* = 9.94%) but were generally high. More specifically, 29 of the 32 TD children chose the shape-matching item more than what was expected by a 50% preference, and eight of the 32 children demonstrated extremely high PCCs that were greater than or equal to 80% across all completed trials. (We adopted 50/50 (i.e., 50%) as a baseline comparison point because each child was faced with a binary choice outcome (shape or material), after removing no responses, across all 16 trials. In this case, since we are concerned with the shape-matched choice as our outcome, a value above 50% would indicate a preference to shape, and a value below 50% would indicate a preference to material. Moreover, using 50% as a baseline is consistent with how researchers typically define the *t*-test against chance, where the aggregated shape choices of LT and TD children are compared against a value of 0.50 (see Perry and Kucker [4] for an example). Additionally, we chose 80% as a benchmark for an *impressive* bias to shape because a child whose shape choice frequency exceeded this value would have had to have chosen the shape choice for the large majority of trials, regardless of set and regardless of no responses. For example, if a child completed two sets worth of data (i.e., eight trials), then the child would have to choose a shape for seven out of eight trials to have a shape choice percentage greater than 80%. Specifically, this would mean the child chose a shape perfectly for one set and got at least three out of four shape choices for the next set). Only two TD children showed slight material biases with PCCs of 40.00% and 46.15%.

The results for all LT children are similarly reported in the right-hand columns of Table 1, and as can be seen only 18 of the 32 LT children performed better than what was expected by a 50% preference: viz., PCCs > 50% and *c*-values < 0.20. The PCCs were also more varied (*absolute median deviation* = 13.20%) with six LT children exhibiting low tendencies to select the shape-matched item (PCCs < 40%) and, remarkably, two LT children (case #’s 46 and 62) exhibiting impressive shape choice frequencies with computed PCCs equal to 93.75% and 87.50%, respectively. These two children were the only LT children with PCCs above 80% across all completed trials. Finally, 10 children showed slight to moderate material biases as their PCCs were less than 50% (*min* = 31.25%). None of the *c*-values for these children were less than 10% when evaluating material preference using a randomization test.

In summary, these person-centered analyses allowed us to go beyond the original *t*-test results and identify the individual children from each group who performed better than chance in the NNG task. Nearly all the TD children chose the shape item at rates higher than a 50% preference, whereas only a slight majority of LT children chose the shape item at rates that exceeded 50%. Moreover, two of the LT children performed on par with or better than most of their TD peers. The PCCs for both groups revealed heterogeneity in shape choice frequencies, with greater variability noted for the LT children compared to the TD children.

### 3.2. Group Comparisons with Persons

Do TD children differ from LT children in terms of their *average* tendency to choose shape matches? Perry and Kucker [4] performed a between-subjects ANOVA to answer this question, and their results revealed a statistically significant, higher average shape choice for the TD children. (Although the TD and LT children were matched on age and gender, Perry and Kucker treated the two groups as independent in each of their statistical analyses. To be consistent with their approach, we also treated the groups as independent. The matched pairs of children are presented side-by-side in Table 1). From a person-centered perspective, a related question based on the children’s individual responses can be asked; namely, “*does the total of the individual shape choice responses for the TD children exceed the total responses for the LT children?*” The advantages of asking this question will again entail focusing upon tallies of individual responses rather than means and variances while not losing sight of the differences in heterogeneity within the two groups of children.

**Figure 4:**
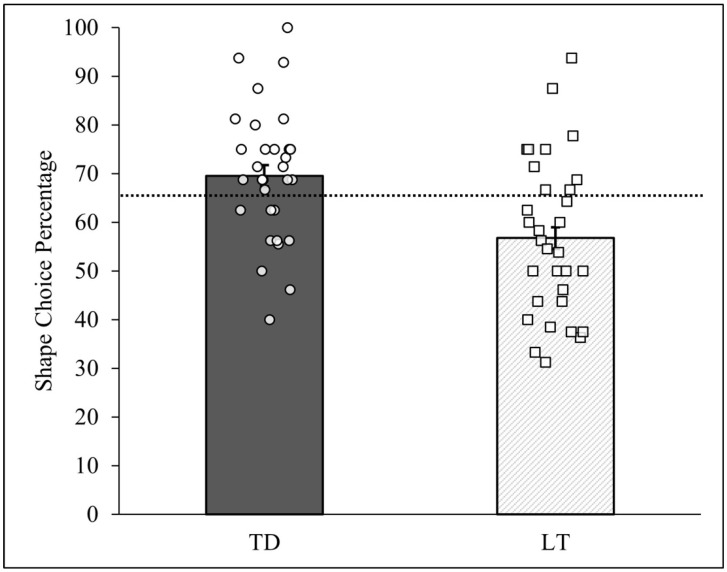
Person-centered shape choice results by talking status. Note: Group-level PCCs for TD and LT children are reported as bars. Each circle represents one of the 32 TD children, and each square represents one of the 32 LT children. The dashed line represents the median shape choice percentage (PCC*_mdn_* = 65.48%) across all 64 children. The error bars represent the standard deviations of the 10,000-iteration randomized PCCs for each trial and group.

The pattern analysis described above can also tally responses across all of the children, thus generating group-level results. Figure 4 reports the two PCCs for the computed shape preference across all TD and LT children and standard deviations (as error bars) derived from the randomization tests for each group. Each child’s PCC taken from Table 1 is also shown in the figure. As can be seen, across all TD children the shape-matched item was chosen for 325 of the 467 completed trials (PCC = 69.59%, *c* < 0.0001), while across all LT children the shape-matched item was chosen for 260 of the 458 completed trials (PCC = 56.77%, *c* = 0.001). These group-level PCCs correspond to the observed mean proportions (0.70 and 0.57, respectively) reported and tested by Perry and Kucker [4]. The group difference in shape-matched choices between the TD and LT children was thus equal to +/−12.82%. This difference was itself evaluated using a randomization test based on the differences between the 10,000 PCCs generated from the TD and LT group randomization tests (viz., PCC_diff_ = PCC_TD_ − PCC_LT_). The results revealed that only one of these differences equaled or exceeded +/−12.82%, thus yielding a *c*-value = 0.0001.

In summary, the person-centered analysis, like Perry and Kucker’s [4] ANOVA, revealed that the TD children chose the shape-matched item at a frequency higher than their LT peers. This difference was moreover judged as not plausibly due to physical chance alone. Importantly, the heterogeneity within each group of children was again made clear in the analysis as their individual PCCs were plotted in the bar graph. The two LT children, case #’s 46 and 62, who exhibited unusually high shape choice frequencies that were greater than most of the TD children, were also plainly visible in the figure. Despite the group-level nature of the analysis, then, the individual performance of each child was not lost from sight. 

### 3.3. Mixed-Effects and Person-Centered Approaches

The percentage of shape choices in our two previous analyses were found to be heterogeneous in both groups of children, though more so in the late-talking children. Will the addition of another variable to the analysis capture this unexplained variability? Perry and Kucker [4] sought to answer this question by creating a novel variable (shape-based vocabulary) that informed them whether the child’s shape-based vocabulary was above, equal to, or below their expectations based upon each child’s residual object noun vocabulary size. Going beyond the single-factor ANOVA, they used a mixed-effects multiple regression analysis to examine the impact of this moderator variable on the relationship between group membership (TD vs. LT) and the shape choice PCCs examined in the analyses above. The result was statistically significant, and follow-up tests revealed that for the TD children, higher shaped-based vocabulary was associated with a greater tendency of choosing shape matches during the NNG task. For the LT children, shape-based vocabulary was not associated with an increased tendency to choose the shape-matching item. However, excluding two outliers from the analysis showed no interaction, only a main effect of language group.

As with the group-level analyses above, the person-centered approach generates questions that revolve around the children themselves rather than around means and variances, allowing for a better way to examine outliers without requiring their removal. Two relevant findings from the analyses above are the following: (1) two LT children showed extreme tendencies to the shape-matched item (PCCs > 85%), and (2) LT children showed greater heterogeneity in their novel noun generalization compared to the TD children. By applying the person-centered approach, some additional questions can be assessed. For instance, can the shape-based vocabulary variable created by Kucker and Perry help explain these two relevant findings? Particularly, do the two LT children that showed a strong tendency to select the shape-matching items also have especially large shape-based vocabularies? If differences in the size of children’s shape-based vocabularies helps to explain differences in the tendency of showing a bias to shape choices among TD children [63], does it also do so for LT children? Consequently, the two LT children that demonstrated a high tendency in selecting the shape-matching item were first examined.

The analyses to address these additional person-centered questions and the two unusual LT children with high shape choices begin by first binning the shape-based vocabulary variable, in order to create three categories for the analysis. Consistent with Perry and Kucker’s [4] description (e.g., a positive residual score suggests the child’s shape-based vocabulary was above expectations, given the group mean), the residual scores were used to bin children into three groups with nearly equal sample sizes: below expectation (*n* = 21; shape residuals [−0.721, −0.038]), approximately equal to expectation (*n* = 22; shape residuals [−0.031, 0.021]), and above expectation (*n* = 21; shape residuals = [0.022, 0.284]). As no child’s residual score was exactly equal to zero, values close to zero were considered as “approximately equal to expectation”. 

With our binned scores in hand, the two LT children with strong tendencies for selecting the shape-matching items can now be further considered. Case #46, whose shape choice PCC was equal to 93.75%, was found to be included in the below-expectation group for shape-based vocabulary, whereas case #62 (shape choice PCC = 87.50%) was found to be included in the approximately equal group. Visual examination of the results for all 32 LT children did not reveal a clear relationship between the tendency of shape-matching choices and shape-based vocabulary variables. To examine potential dependencies between shape-based vocabulary and group (TD vs. LT), the two categorical variables were first crossed to create six groups of children. Harris [67] showed that moderated effects (i.e., interactions) can be investigated efficiently by creating such a grouping variable from crossed combinations of independent variables in a study. 

**Figure 5:**
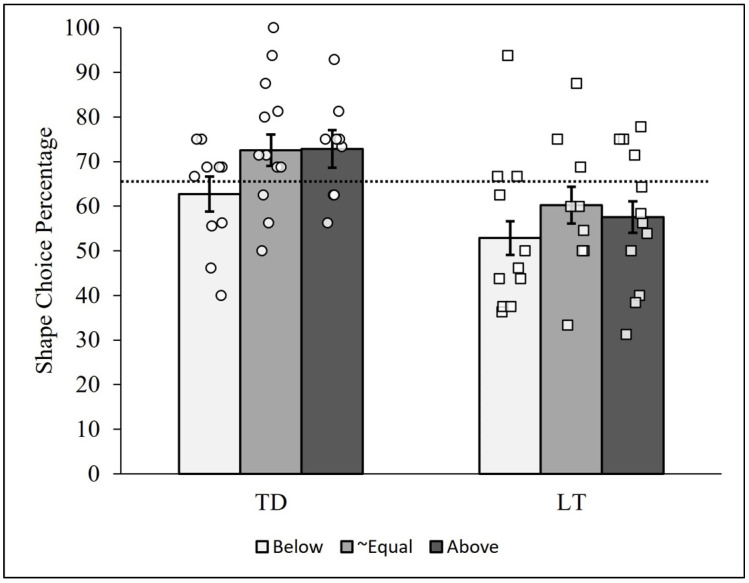
Person-centered shape choice results between talking status and shape residuals. Note: Bars represent the group-level shape choice PCCs for the typically developing (TD) and late-talking (LT) children below, approximately equal to, and above expected shape residual scores. Each circle represents a TD child, and each square represents an LT child. The dashed line represents the median shape choice percentage (PCC*_mdn_* = 65.48%) across all 64 children. The error bars represent the standard deviations of the 10,000-iteration randomized PCCs for each trial and group.

The pattern analysis used in the first two analytic examinations comparing the *t*-test and ANOVA was then conducted using each of these six groups of children. The resulting PCCs are reported in Figure 5. As can be seen, the TD children typically showed more shape-matching choices than the LT children, as already reported above. All groups showed a great deal of heterogeneity as well, with a potentially influential case in the LT/below-expectation group (PCC = 93.75, case #46). Importantly, the overall patterns of PCCs for the TD and LT children (i.e., the relative bar heights in the figure) did not differ markedly across the below-expectation, approximately equal, and above-expectation shape vocabulary groups. The PCC for the TD children was higher than the PCC for the LT children in all three groups, and the differences were similar in magnitude. In other words, the pattern of results in Figure 5 did not reveal visually compelling evidence of an interaction (i.e., a moderation effect) between the two variables.

Specific comparisons between PCCs computed from all responses for each group of children supported this conclusion. The TD children in the below-expectation group (PCC = 62.76%) showed overall fewer shape choices than their approximately equal (PCC = 72.54%) and above-expectation (PCC = 72.87%) counterparts. The *c*-values comparing the below-expectation to the approximately equal and above-expectation group PCCs were low (viz., 0.04 and 0.03, respectively), whereas the *c*-value based on comparing the approximately equal and above-expectation PCCs was high (0.95). 

For the LT children, the below-expectation group (PCC = 52.87%) similarly showed overall fewer shape choices than the approximately equal (PCC = 60.29%) and above-expectation (PCC = 57.58%) groups. The *c*-values comparing pairs of group-level PCCs for these three groups, however, were not very low (viz., >0.18). Removing the influential case from the LT/below-expectation group reduced the PCC to 48.23%, resulting in an even more parallel pattern of PCCs and *c*-values between the TD and LT groups; specifically, the *c*-values comparing the below-expectation group to the approximately equal and above-expectation LT groups were reduced to 0.04 and 0.08, respectively. These results therefore indicate stronger support for a lack of interaction between the binned shape-based vocabulary and group variables.

In summary, the person-centered analyses revealed that the two unusual LT children with strong tendencies of selecting the shape-matching items did not have above-expectation shape-based vocabularies, as one might anticipate based on theory. Instead, these two children (case #’s 46 and 62) had below- or approximately-equal-to-expectation vocabularies. Considering all children, heterogeneity in the individual shape choice percentages was still highly visible in each of the six groups plotted in Figure 5. With or without the influential case in the LT/below-expectation group, the patterns of overall PCCs for the TD and LT children in the figure were moreover highly similar, indicating that the addition of the shape-based vocabulary variable to the analysis unfortunately did not help to further explain the heterogeneity (i.e., variability) in the tendency to choose the shape-matching items.

### 3.4. Random Effects and Person-Centered Approaches

Within the NNG paradigm, the sets of objects were randomized so that children did not get the same sets in the same order. For example, one child may have received the items ordered as zup, mip, kiv, and fum, whereas another child may have received the items ordered as mip, kiv, fum, and zup. In this way, each child judged each of the items four times, but potential item and order effects were controlled. Nonetheless, there is a question of if variation across the zup, fum, kiv, and mip items is substantial and potentially important. To account for this, Perry and Kucker [4] included a random intercept of item in their mixed-effects regression analysis, though its impact was nonsignificant in each of the models tested (*p*-values > 0.20). As with its emphasis on individual children, the person-centered approach emphasizes responses to individual items as well. Consequently, an additional question to be asked is “*do particular items reveal a propensity toward shape choices, and is this propensity found equally among TD and LT children?*”.

**Figure 6:**
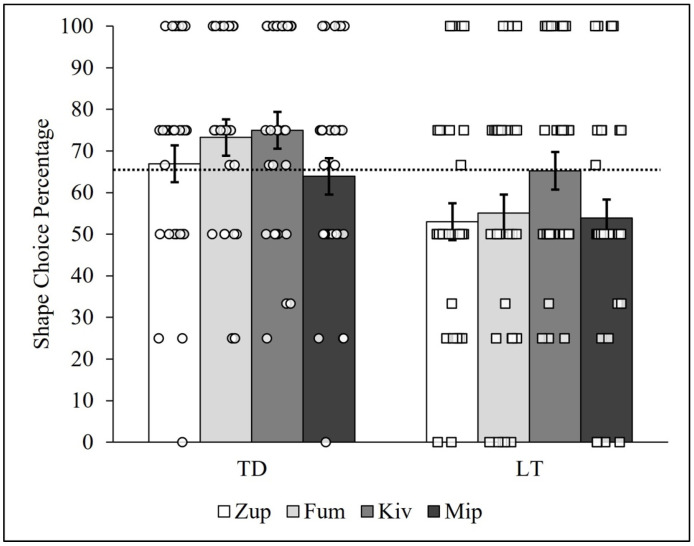
Typically developing and late-talking children’s shape choice PCCs by item. Note: Novel noun generalization PCCs for TD and LT children. Circles represent TD children, whereas squares represent LT children. The dashed line represents the median shape choice percentage (PCC*_mdn_* = 65.48%) across all 64 children. The error bars represent the standard deviations of the 10,000-iteration randomized PCCs for each trial and group.

The same type of pattern analyses utilized previously (see Figure 2 and Figure 3) were conducted to determine whether the individual TD or LT children chose the shape-matched item at a higher rate for certain items (e.g., zup) compared to others (e.g., fum, kiv, and mip). Accordingly, the data were re-organized so that all four trials of each item set were analyzed together, for a total of four computed PCCs for both groups of children. Figure 6 shows the general results of the percentage of shape choices (i.e., NNG PCCs) between the TD and LT children for the zup, fum, kiv, and mip items in the bar graphs, along with plots of the individual children’s NNG PCCs. 

The TD children outperformed the LT children on each of the four corresponding items, choosing the shape-matched item with frequencies above 60% for each. As can be seen in the figure, the TD children’s lowest percentage of shape-matching choices (PCC = 63.93%, *c* = 0.001) was observed for the mip item. Notably, variation between items was observed, as the differences between the TD children’s NNG PCCs for kiv and mip (PCC_diff_ = 11.07%, *c* = 0.07) and for fum and mip (PCC_diff_ = 9.35%, *c* = 0.15) were less plausibly explainable as due to physical chance. The difference between the NNG PCCs between the zup and mip sets (PCC_diff_ = 3.01%, *c* = 0.66) was more plausibly explained as having arisen from physical chance.

The NNG PCCs for the LT children were similar in magnitude for the zup, fum, and mip items. Interestingly, the LT children’s highest percentage of shape-matching choices (PCC = 65.22, *c* < 0.001) occurred for the kiv item and was nearly equal to the median PCC value (65.48%) for both groups and as well as the zup and mip PCCs for the TD children (66.94% and 63.93%, respectively, *c*’s < 0.001; see Figure 5). The differences between LT children’s NNG PCCs for kiv and mip (PCC_diff_ = 11.37, *c* = 0.08), kiv and fum (PCC_diff_ = 10.17%, *c* = 0.09), and kiv and zup (PCC_diff_ = 12.23%, *c* = 0.05) were less plausibly explainable by physical chance. In summary, both the TD and LT children showed heterogeneity in shape choices between the four items, and the LT children showed a relatively high percentage of shape choices for the kiv item that was nearly equal to the percentage of shape choices of TD children for the zup and mip items. The TD children by comparison chose the shape over the material at a frequency greater than 60% for each of the four items.

In summary, the person-centered analyses focusing on the items in the NNG task yielded novel results. Specifically, the patterns of responses suggest there may be item effects present for the LT children, particularly the kiv item, that exceed physical chance. In other words, something about the kiv object may have caused the LT children to attune to its shape more than the material of which it was made. Similarly, exploratory analyses of trial order effects also found high heterogeneity across the groups of children (see Supplemental Files). Indeed, prior work has suggested a critical role for item-specific features more generally [68] and order effects of items [69] in children’s word learning. Together with the item-level analysis, this lays the foundation for future work with LT children. 

## 4. Discussion

Recent advances in statistical methodologies have provided developmental and language development researchers with a remarkably diverse and powerful set of analytical tools for testing their empirical hypotheses. The re-analysis of Perry and Kucker’s [4] data above shows that person-centered methods are an important addition to this ever-growing list of tools as they can be used to address questions about group differences as well as unique questions about individual participants (see also [8]). For example, how many individuals in a group match expectation with regard to their responses, and are there differences in variability of responses (i.e., heterogeneity) between groups? Are there individuals like Suzy and Charlie (two LTs, one of whom blooms late and the other who has persisting delays), and how can their differences be understood? With person-centered methods such questions revolve around the individual responses of participants in any given study. Unlike ANOVA, multiple regression, and other traditional analyses, these methods do not require the computation of a single mean, variance, or covariance. This fact was demonstrated here by examining individual responses tallied in the context of a predicted pattern to address hypotheses about particular children, and raw responses similarly tallied across children to address group-level hypotheses. 

Focusing on individual responses in Perry and Kucker’s [4] study created an avenue to also examine children’s behaviors toward particular stimuli. Results from these analyses revealed that LT children showed a qualitatively different response pattern toward one particular set of items in this study (the kiv). This unique effect was missed by the mixed-effects regression analyses based on aggregate statistics. Should the kiv item be considered as a potential methodological confound, or is there something to learn theoretically by examining it more closely? What additional items, if any, yield similar patterns of novel noun generalization between the TD and LT children? Such questions can open the door to further theoretical developments and exploration of how individual stimuli may impact models such as that by Smith et al. [62]. Focusing on individuals can also generate important questions about research design. Using person-centered methods, for example, Grice and colleagues [18] discovered a methodological confound which had gone undetected in an established research paradigm used by evolutionary cognitive psychologists. For Perry and Kucker’s [4] study, are the number of responses collected from each child sufficient for determining whether or not learning had occurred? Should each child be assessed twice using the same techniques to address individual reliability, thereby buttressing the conclusions? After all, as advocates of single-subject research designs have long pointed out, the strongest claim of replicability is made at the level of the individual, not at the level of the aggregate (e.g., see [70,71,72]). Answering such questions and considering the related issues would strengthen the conclusions drawn from the current results.

With regard to implications for the study of language development more generally, the results here show the utility of considering individual-level data when predicting abilities for an individual child as well as exploring patterns of performance across a highly variable group of children. For decades, language researchers have grappled with variations of the question “which children will progress on their own and which will continue to lag behind their peers?” For instance, how do we track LT children’s vocabulary growth to determine who will “catch up” [5,38], and how can we predict outcomes of children with DLD or cochlear implants [73,74,75]? The answers have a common thread—individual-level data. 

While our standard analytical approaches can offer some insight and do provide critical foundations for advancing our understanding of language development, tools such as those demonstrated here can allow for a deeper dive into current data sets and set up a new perspective for future data. Importantly, such an approach is also flexible, allowing for the inclusion of a complex array of individual factors that may be driving behavior (see [8]). Indeed, the analysis here is not limited solely to the three variables explored (late talker status, shape vocabulary, item effects), but as shown in the Supplemental Materials, can illuminate the impacts of trial order and fatigue effects, which are particularly relevant when testing young infants. That is, person-centered approaches like those analyzed here allow researchers to see new patterns that may give beneficial insight into the validity of the methods themselves as well as critical views of the individual child’s performance.

Regarding the benefits as they pertain to speech language pathologists or clinically oriented language researchers, let us return to the case of Charlie one final time. Suppose the data from Perry and Kucker [4] had been collected after the completion of an intervention aimed at bolstering late-talking children’s attention to shapes when learning novel nouns. In such a scenario, the person-centered analyses would have readily identified two ‘Charlies’ who would have shown highly successful results in support of the hypothetical intervention. Furthermore, had competing interventions been tested, the person-centered analyses would readily allow for direct comparisons of the competing interventions (see [76]). Such information would be of immense benefit in helping explain other late-talking children.

In closing, much like using both idiographic and nomothetic approaches to provide a more complete understanding of infant behavior [77], researchers can use person-centered methods like those above as complements to traditional statistical analyses because they possess a number of distinct and helpful properties. First, as demonstrated above, they lead to questions that are not normally considered when using traditional methods. Second, they are easy to use and yield results that are easy to understand. The Percent Correct Classifications index is essentially a universal index of effect size, and the *c*-value is a distribution-free statistic that eschews the common assumptions underlying the traditional *p*-value in NHST. Third, while interval- and ratio-scaled observations can be analyzed with person-centered methods (e.g, [20,21,78]), the assumption of continuous, quantitative measurement is not necessary. Perry and Kucker [4] measured children’s responses in a binary fashion and noted that *t*-tests and ANOVAs are not optimal for these categorical outcome observations (see also [79]). (With regard to binary variables, the classification of children as late-talking or typically developing is worth further consideration. The cut-off for distinguishing between the two types is arbitrary and there is growing evidence to suggest that language abilities represent more of a continuum with LT children simply at the lower end of the spectrum compared to TD children. Indeed, in other analyses of this same set of data, there is very little difference in results if a continuous measure of vocabulary percentile is used rather than a dichotomous classification (LT vs. TD; [80]; see also [43,50,81]). Given this work, we do not anticipate a meaningful difference in results based on how children are categorized. A key point is children with lower language abilities for their age show incrementally different behaviors than those with stronger language skills). The person-centered methods used above are optimal for such data as they do not rely upon the assumption of continuous, quantitative measurement. Furthermore, when the data are binary, such as in the current example, the only assumptions which must be met by the researcher are identical to those which must be met for the binomial test. (The three assumptions of the binomial distribution are as follows: (1) one outcome for each trial, (2) each trial has the same probability of success, and (3) each trial is mutually exclusive or independent of the other trials). Finally, by adopting such simple, clear, and assumption-free methods, developmental and language development researchers can devote more intellectual resources toward building explanatory theories that accurately account for the causes underlying the behaviors of children in their studies and beyond.

## Figures and Tables

**Table 1:** Typically developing and late-talking children shape choice results.

	Typically Developing				Late Talking		
Case #	Trials	PCC	*c*-Value	Case #	Trials	PCC	*c*-Value
1	16	56.25	0.40	33	16	75.00	0.04
2	16	68.75	0.10	34	16	62.50	0.23
3	12	66.67	0.19	35	16	43.75 *	0.78
4	15	40.00 *	0.85	36	11	36.36 *	0.89
5	12	75.00	0.07	37	16	56.25	0.40
6	14	71.43	0.09	38	16	50.00 *	0.60
7	15	73.33	0.06	39	13	46.15 *	0.71
8	16	68.75	0.10	40	15	60.00	0.30
9	14	92.86	0.001	41	15	33.33 *	0.94
10	16	68.75	0.10	42	12	58.33	0.38
11	16	75.00	0.04	43	15	60.00	0.30
12	16	68.75	0.11	44	16	50.00 *	0.59
13	16	75.00	0.04	45	10	40.00 *	0.83
14	16	62.50	0.22	46	16	93.75	<0.0001
15	8	62.50	0.37	47	13	53.85	0.49
16	6	100.00	0.02	48	14	64.29	0.21
17	16	75.00	0.04	49	16	75.00	0.04
18	16	75.00	0.04	50	16	43.75 *	0.77
19	16	62.50	0.22	51	14	71.43	0.09
20	16	68.75	0.10	52	16	50.00 *	0.60
21	13	46.15 *	0.71	53	6	66.67	0.34
22	9	55.56	0.50	54	11	54.55	0.50
23	15	80.00	0.02	55	13	38.46 *	0.86
24	16	56.25	0.40	56	16	31.25 *	0.96
25	16	50.00 *	0.60	57	16	37.50 *	0.89
26	16	93.75	<0.0001	58	9	77.78	0.09
27	16	68.75	0.10	59	16	75.00	0.04
28	14	71.43	0.09	60	16	68.75	0.11
29	16	56.25	0.41	61	16	50.00 *	0.60
30	16	87.50	0.002	62	16	87.50	0.002
31	16	81.25	0.01	63	16	37.50 *	0.90
32	16	81.25	0.01	64	15	66.67	0.16
Totals	467	69.59	<0.0001	Totals	458	56.77	0.002

Note: Trial numbers vary because some children did not complete all sixteen trials. The chance-value (*c*-value) is from a randomization test with 10,000 iterations. PCC = Percent Correct Classifications index. * Denotes a child’s PCC that was equal to or less than a 50/50 preference.

## Data Availability

The data and all materials are publicly available via the Open Science Framework: https://osf.io/b83hz/, accessed on 7 August 2024.

## Supplementary Materials

The following supporting information can be downloaded at: https://www.mdpi.com/article/10.3390/bs14080708/s1, Figure S1: Typically Developing and Late Talking Children’s Shape-Choice PCCs by Trial; Figure S2: Person-Centered Shape-Choice Results Split by Trial Order and Talker Status; Figure S3: Person-Centered Shape-Choice Results Split by Trial Order, Talker Status, and Shape Residual Categorization; Table S1: Trial Order Effects in Typically Developing and Late Talking Children’s Shape-Choice PCCs; Table S2: TD Children’s Shape-Choice Results Separated by Trial Order; Table S3: LT Children’s Shape-Choice Results Separated by Trial Order. References [82,83] are cited in the Supplementary Materials.

## References

[1] Bates E., Dale P.S., Thal D. (1995). Individual differences and their implications for theories of language development. Handb. Child Lang..

[2] Gershkoff-Stowe L., Smith L.B. (2004). Shape and the first hundred nouns. Child Dev..

[3] McCullough K.C., Bayles K.A., Bouldin E.D. (2019). Language performance of individuals at risk for mild cognitive impairment. J. Speech Lang. Hear. Res..

[4] Perry L.K., Kucker S.C. (2019). The heterogeneity of word learning biases in late-talking children. J. Speech Lang. Hear. Res..

[5] Perry L.K., Kucker S.C., Horst J.S., Samuelson L.K. (2022). Late bloomer or language disorder? Differences in toddler vocabulary composition associated with long-term language outcomes. Dev. Sci..

[6] Dollaghan C. (1998). Late talker or SLI?: The story of Jay X. Semin. Speech Lang..

[7] McManus R.M., Young L., Sweetman J. (2023). Psychology is a property of persons, not averages or distributions: Confronting the group-to-person generalizability problem in experimental psychology. Adv. Methods Pract. Psychol. Sci..

[8] van der Gaag M.A. (2023). A person-centered approach in developmental science: Why this is the future and how to get there. Infant Child Dev..

[9] Gordon K.R. (2019). How mixed-effects modeling can advance our understanding of learning and memory and improve clinical and educational practice. J. Speech Lang. Hear. Res..

[10] McMillan G.P., Cannon J.B. (2019). Bayesian applications in auditory research. J. Speech Lang. Hear. Res..

[11] Oleson J.J., Brown G.D., McCreery R. (2019). The evolution of statistical methods in speech, language, and hearing sciences. J. Speech Lang. Hear. Res..

[12] Paulon G., Reetzke R., Chandrasekaran B., Sarkar A. (2019). Functional Logistic Mixed-Effects Models for Learning Curves From Longitudinal Binary Data. J. Speech Lang. Hear. Res..

[13] Walker E.A., Redfern A., Oleson J.J. (2019). Linear mixed-model analysis to examine longitudinal trajectories in vocabulary depth and breadth in children who are hard of hearing. J. Speech Lang. Hear. Res..

[14] Byers-Heinlein K., Bergmann C., Savalei V. (2021). Six solutions for more reliable infant research. Infant Child Dev..

[15] Syed M. (2022). Special issue on reliability of infant research. Infant Child Dev..

[16] Davis-Kean P.E., Ellis A. (2019). An overview of issues in infant and developmental research for the creation of robust and replicable science. Infant Behav. Dev..

[17] Frank M.C. (2019). Towards a more robust and replicable science of infant development. Infant Behav. Dev..

[18] Grice J.W., Medellin E., Jones I., Horvath S., McDaniel H., O’lansen C., Baker M. (2020). Persons as effect sizes. Adv. Methods Pract. Psychol. Sci..

[19] Speelman C.P., McGann M. (2020). Statements about the pervasiveness of behavior require data about the pervasiveness of behavior. Front. Psychol..

[20] Arocha J.F. (2020). Scientific realism and the issue of variability in behavior. Theory Psychol..

[21] Beechey T. (2023). Ordinal Pattern Analysis: A Tutorial on Assessing the Fit of Hypotheses to Individual Repeated Measures Data. J. Speech Lang. Hear. Res..

[22] Erisman M.C., Blom E. (2020). Reading outcomes in children with developmental language disorder: A person-centered approach. Autism Dev. Lang. Impair..

[23] de Klerk M., Veen D., Wijnen F., de Bree E. (2019). A step forward: Bayesian hierarchical modelling as a tool in assessment of individual discrimination performance. Infant Behav. Dev..

[24] Sayette M.A., Goodwin M.E., Creswell K.G., Esmacher H.J., Dimoff J.D. (2022). A Person-Centered Analysis of Craving in Smoking-Cue-Exposure Research. Clin. Psychol. Sci..

[25] Valentine K.D., Buchanan E.M. (2013). JAM-boree: An application of observation oriented modelling to judgements of associative memory. J. Cogn. Psychol..

[26] Valentine K.D., Buchanan E.M., Scofield J.E., Beauchamp M.T. (2019). Beyond p values: Utilizing multiple methods to evaluate evidence. Behaviormetrika.

[27] Nketia J., Amso D., Brito N.H. (2021). Towards a more inclusive and equitable developmental cognitive neuroscience. Dev. Cogn. Neurosci..

[28] Zuo X.N., Xu T., Milham M.P. (2019). Harnessing reliability for neuroscience research. Nat. Hum. Behav..

[29] Ferguson C.J. (2009). Is psychological research really as good as medical research? Effect size comparisons between psychology and medicine. Rev. Gen. Psychol..

[30] Funder D.C., Ozer D.J. (2019). Evaluating effect size in psychological research: Sense and nonsense. Adv. Methods Pract. Psychol. Sci..

[31] Rutledge T., Loh C. (2004). Effect sizes and statistical testing in the determination of clinical significance in behavioral medicine research. Ann. Behav. Med..

[32] Berk R.A., Freedman D.A., Bloomberg T.G., Cohen S. (2003). Statistical assumptions as empirical commitments. Punishment and Social Control.

[33] Hoff P.D. (2009). A First Course in Bayesian Statistical Methods.

[34] Meeden G. (2012). A Bayesian justification for random sampling in sample survey. Pak. J. Stat. Oper. Res..

[35] Meier L. (2022). Random and Mixed Effects Models. ANOVA and Mixed Models: A Short Introduction Using R.

[36] Bornstein M.H., Jager J., Putnick D.L. (2013). Sampling in developmental science: Situations, shortcomings, solutions, and standards. Dev. Rev..

[37] Oakes L.M. (2017). Sample size, statistical power, and false conclusions in infant looking-time research. Infancy.

[38] Rescorla L. (2011). Late talkers: Do good predictors of outcome exist?. Dev. Disabil. Res. Rev..

[39] Bishop D.V., Holt G., Line E., McDonald D., McDonald S., Watt H. (2012). Parental phonological memory contributes to prediction of outcome of late talkers from 20 months to 4 years: A longitudinal study of precursors of specific language impairment. J. Neurodev. Disord..

[40] Catts H.W., Fey M.E., Tomblin J.B., Zhang X. (2002). A longitudinal investigation of reading outcomes in children with language impairments. J. Speech Lang. Hear. Res..

[41] Conti-Ramsden G., Botting N. (2004). Social difficulties and victimization in children with SLI at 11 years of age. J. Speech Lang. Hear. Res..

[42] Hadley P.A., Rice M.L. (1991). Conversational responsiveness of speech-and language-impaired preschoolers. J. Speech Lang. Hear. Res..

[43] Weismer S.E. (2017). Typical talkers, late talkers, and children with specific language impairment: A language endowment spectrum?. Language Disorders from a Developmental Perspective.

[44] Ellis E.M., Thal D.J. (2008). Early language delay and risk for language impairment. Perspect. Lang. Learn. Educ..

[45] Fischel J.E., Whitehurst G.J., Caulfield M.B., DeBaryshe B. (1989). Language growth in children with expressive language delay. Pediatrics.

[46] Girolametto L., Wiigs M., Smyth R., Weitzman E., Pearce P.S. (2001). Children with a history of expressive vocabulary delay. Am. J. Speech-Lang. Pathol..

[47] Paul R., Murray C., Clancy K., Andrews D. (1997). Reading and metaphonological outcomes in late talkers. J. Speech Lang. Hear. Res..

[48] Rescorla L. (2002). Language and reading outcomes to age 9 in late-talking toddlers. J. Speech Lang. Hear. Res..

[49] Rescorla L. (2005). Age 13 language and reading outcomes in late-talking toddlers. J. Speech Lang. Hear. Res..

[50] Rescorla L. (2009). Age 17 language and reading outcomes in late-talking toddlers: Support for a dimensional perspective on language delay. J. Speech Lang. Hear. Res..

[51] Rice M.L., Taylor C.L., Zubrick S.R. (2008). Language outcomes of 7-year-old children with or without a history of late language emergence at 24 months. J. Speech Lang. Hear. Res..

[52] Fernald A., Marchman V.A. (2012). Individual differences in lexical processing at 18 months predict vocabulary growth in typically developing and late-talking toddlers. Child Dev..

[53] Fisher E.L. (2017). A systematic review and meta-analysis of predictors of expressive-language outcomes among late talkers. J. Speech Lang. Hear. Res..

[54] Collisson B.A., Grela B., Spaulding T., Rueckl J.G., Magnuson J.S. (2015). Individual differences in the shape bias in preschool children with specific language impairment and typical language development: Theoretical and clinical implications. Dev. Sci..

[55] Colunga E., Sims C.E. (2017). Not only size matters: Early-talker and late-talker vocabularies support different word-learning biases in babies and networks. Cogn. Sci..

[56] Jones S.S. (2003). Late talkers show no shape bias in a novel name extension task. Dev. Sci..

[57] Jones S.S., Smith L.B. (2005). Object name learning and object perception: A deficit in late talkers. J. Child Lang..

[58] Kucker S.C., Samuelson L.K., Perry L.K., Yoshida H., Colunga E., Lorenz M.G., Smith L.B. (2019). Reproducibility and a unifying explanation: Lessons from the shape bias. Infant Behav. Dev..

[59] Landau B., Smith L.B., Jones S.S. (1988). The importance of shape in early lexical learning. Cogn. Dev..

[60] Perry L.K., Samuelson L.K., Malloy L.M., Schiffer R.N. (2010). Learn locally, think globally: Exemplar variability supports higher-order generalization and word learning. Psychol. Sci..

[61] Samuelson L.K. (2002). Statistical regularities in vocabulary guide language acquisition in connectionist models and 15–20-month-olds. Dev. Psychol..

[62] Smith L.B., Jones S.S., Landau B., Gershkoff-Stowe L., Samuelson L. (2002). Object name learning provides on-the-job training for attention. Psychol. Sci..

[63] Perry L.K., Samuelson L.K. (2011). The shape of the vocabulary predicts the shape of the bias. Front. Psychol..

[64] Grice J.W. (2024). OOM: Observation Oriented Modeling [Computer Software]. https://idiogrid.com/OOM/.

[65] Grice J.W. (2011). Observation Oriented Modeling: Analysis of Cause in the Behavioral Sciences.

[66] Grice J.W. (2021). Drawing inferences from randomization tests. Personal. Individ. Differ..

[67] Harris R.J. (1994). ANOVA: An Analysis of Variance Primer.

[68] Pomper R., Saffran J.R. (2019). Familiar object salience affects novel word learning. Child Dev..

[69] Samuelson L.K., Horst J.S. (2007). Dynamic noun generalization: Moment-to-moment interactions shape children’s naming biases. Infancy.

[70] Horner R.H., Carr E.G., Halle J., McGee G., Odom S., Wolery M. (2005). The use of single subject research to identify evidence-based practice in special education. Except. Child..

[71] Kazdin A.E. (2011). Single-Case Research Designs: Methods for Clinical and Applied Settings.

[72] McReynolds L.V., Kearns K. (1983). Single-Subject Experimental Designs in Communicative Disorders.

[73] Botting N., Faragher B., Simkin Z., Knox E., Conti-Ramsden G. (2001). Predicting Pathways of Specific Language Impairment: What Differentiates Good and Poor Outcome?. J. Child Psychol. Psychiatry Allied Discip..

[74] Peterson N.R., Pisoni D.B., Miyamoto R.T. (2010). Cochlear implants and spoken language processing abilities: Review and assessment of the literature. Restor. Neurol. Neurosci..

[75] Pisoni D.B., Kronenberger W.G., Harris M.S., Moberly A.C. (2017). Three challenges for future research on cochlear implants. World J. Otorhinolaryngol.-Head Neck Surg..

[76] Grice J.W., Barrett P.T., Cota L., Taylor Z., Felix C., Garner S., Medellin E., Vest A. (2017). Four bad habits of modern psychologists. Behav. Sci..

[77] Pérez-Edgar K., Vallorani A., Buss K.A., LoBue V. (2020). Individual differences in infancy research: Letting the baby stand out from the crowd. Infancy.

[78] Grice J.W., Craig D.P.A., Abramson C.I. (2015). A simple and transparent alternative to repeated measures ANOVA. Sage Open.

[79] Jaeger T.F. (2008). Categorical data analysis: Away from ANOVAs (transformation or not) and towards logit mixed models. J. Mem. Lang..

[80] Kucker S.C., Seidler E. (2023). The timescales of word learning in children with language delays: In-the-moment mapping, retention, and generalization. J. Child Lang..

[81] Thal D.J., Bates E., Goodman J., Jahn-Samilo J. (1997). Continuity of language abilities: An exploratory study of late-and early-talking toddlers. Dev. Neuropsychol..

[82] Fidler F., Loftus G.R. (2009). Why figures with error bars should replace p values: Some conceptual arguments and empirical demonstrations. Z. Psychol./J. Psychol..

[83] Tukey J.W. (1977). Exploratory Data Analysis.

