# Supplementary material for: Capturing the Heterogeneity of Word Learners by Analyzing Persons
Source: Behav Sci (Basel). 2024 Aug 13;14(8):708. doi: 10.3390/bs14080708 (PMC11351650; doi:10.3390/bs14080708)
Supplement: Supplementary file 1 [file behavsci-14-00708-s001.zip › behavsci-3103819-supplementary.pdf]

behavioral sciences

MDPI

Supplementary Materials

Exploratory Results: Assessing Trial Order Effects

The same concatenated pattern analysis performed previously was conducted to determine whether or not there were differences between the TD and LT infants' shape-choice across each of the 16 trials. This specifically is examining trial order effects, such as the possibility of fatigue as the task wears on, or improvement as the child undergoes more and more trials. Instead of computing the PCC for the percentage of shape choices for one infant (e.g., TD Child #2) across all 16 trials, we computed the percentage of shape choices for all infants at trial 1, then trial 2, and so forth to trial 16, yielding a total of 16 PCCs (and *c*-values) for both the TD and LT children. The complete results of the 16 PCCs and *c*-values for both groups of kids can be found in Table S1.

Table S1.
Trial Order Effects in Typically Developing and Late Talking Children's Shape-Choice PCCs

	TD		LT		TD – LT	
	PCC	*c*-value	PCC	*c*-value	PCC_{diff}	*c*-value
Trial 1	72.41	0.003	45.16*	0.70	27.25	0.03
Trial 2	73.33	0.004	60.00	0.11	13.33	0.25
Trial 3	66.67	0.02	55.17	0.30	11.49	0.38
Trial 4	73.33	0.003	42.86*	0.81	30.48	0.02
Trial 5	86.21	< 0.001	51.72	0.43	34.48	0.004
Trial 6	80.00	< 0.001	63.33	0.05	16.67	0.17
Trial 7	65.52	0.06	53.33	0.30	12.18	0.38
Trial 8	76.67	0.001	46.43*	0.70	30.24	0.02
Trial 9	67.74	0.02	64.29	0.06	3.46	0.71
Trial 10	60.00	0.11	58.62	0.18	1.38	0.90
Trial 11	55.56	0.30	53.85	0.30	1.71	0.90
Trial 12	57.14	0.19	52.00	0.43	5.14	0.71
Trial 13	66.67	0.03	76.67	0.001	-10.00	0.38
Trial 14	65.52	0.06	51.61	0.43	13.90	0.26
Trial 15	68.97	0.009	65.38	0.05	3.58	0.70
Trial 16	75.86	0.001	67.86	0.02	8.00	0.53

Note. The chance-value (*c*-value) is from a randomization test with 10,000 iterations.
TD – LT = the difference between typical developing (TD) and late talking (LT) shape choice PCCs at each trial. A positive value indicates the TD children had a higher shape-choice PCC. A negative value indicates the LT children had a higher shape-choice PCC.
PCC = Percent Correct Classifications index.
*Denotes a PCC that was equal to or less than a 50/50 preference.

Looking at Figure S1 below, trial order effects do seem to be present within these data. Specifically, the TD infants had a preference to shape at rates above the median for 6 out of the first 8 trials (i.e., Trials 1 – 8), whereas this was only true for 1 trial during the last 8 (i.e., trials 9 – 16). By comparison, the LT children's shape choice frequencies fell around 50% during 5 of the first 8 trials, but the shape-choice PCCs (i.e., NNG PCCs) strengthened slightly during the last half. That is, TD children appear to be selecting shape and lower rates as the task went on (perhaps due to fatigue), whereas the LT children improved (perhaps due to learning effects).

To further assess the trial order effects' impact on the data we re-conducted the *Concatenated Pattern Analyses* for the TD and LT children separated by the first 8 trials and then the last 8 trials. Additionally, we include the same "mixed-model" analyses conducted in our primary results for the TD and LT infants who's

shape residual category was below, nearly equal to, and above expectations during the first 8 trials and last 8 trials.

Figure S1. Typically Developing and Late Talking Children's Shape-Choice PCCs by Trial

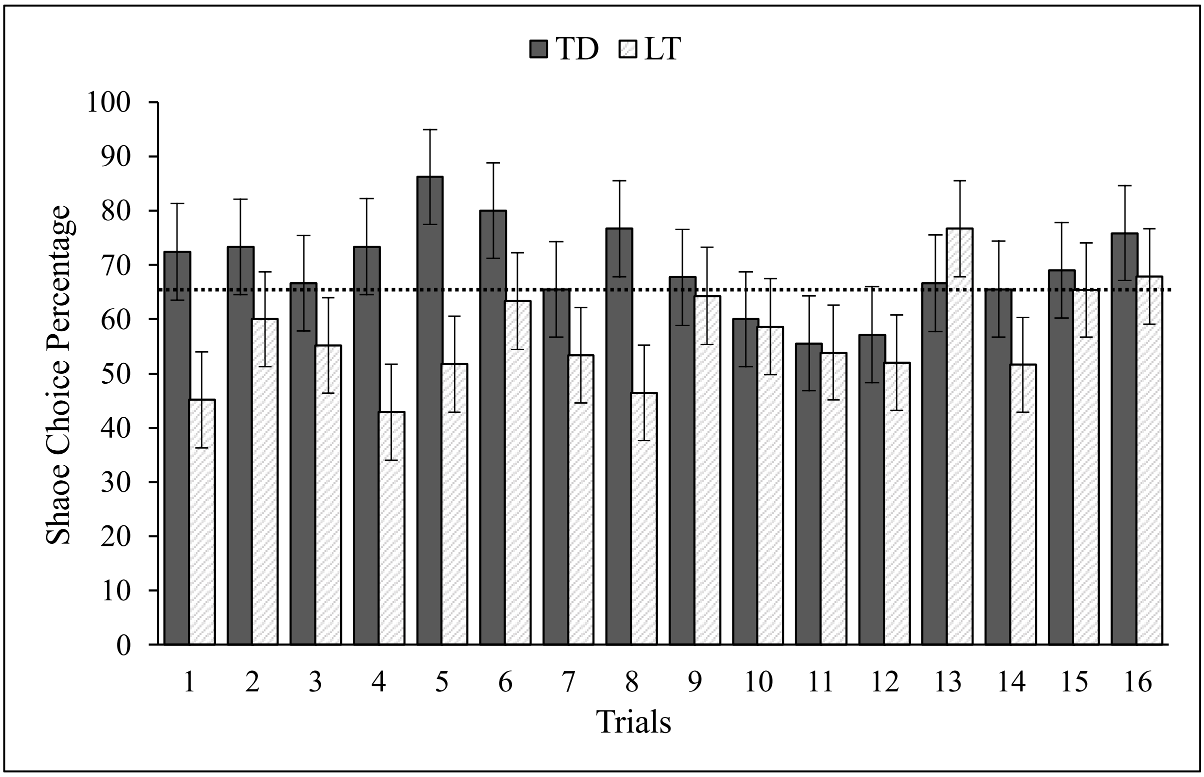

Note. Group-level PCCs for TD and LT children are reported as bars. The dashed line represents the median shape choice frequency (PCC_{mdn} = 65.48%) across all 64 children. The error bars represent the standard deviations of the 10,000-iteration randomized PCCs for each trial and group.

Re-evaluating the Person-Centered Shape Preference in Consideration of Trial Order Effects

The complete individual results for the person-centered shape choice frequency analyses for TD and LT children during the first 8 and last 8 trials can be found in Tables S2 and S3 below, but for brevity we primarily rely upon graphical interpretations [82,83] of the group-level PCCs. Looking at Figure S2, the TD infants, as a group, have a stronger preference to shape during the first 8 trials (PCC = 74.36%, $c < .001$) than during the last 8 (PCC = 64.81%, $c < .001$) trials. Moreover, the difference between the TD infants' shape-choice PCC for the last 8 and first 8 trials (PCC_{diff} = -9.55%) could not plausibly be explained by physical chance ($c = .03$).

Figure S2. Person-Centered Shape-Choice Results Split by Trial Order and Talker Status

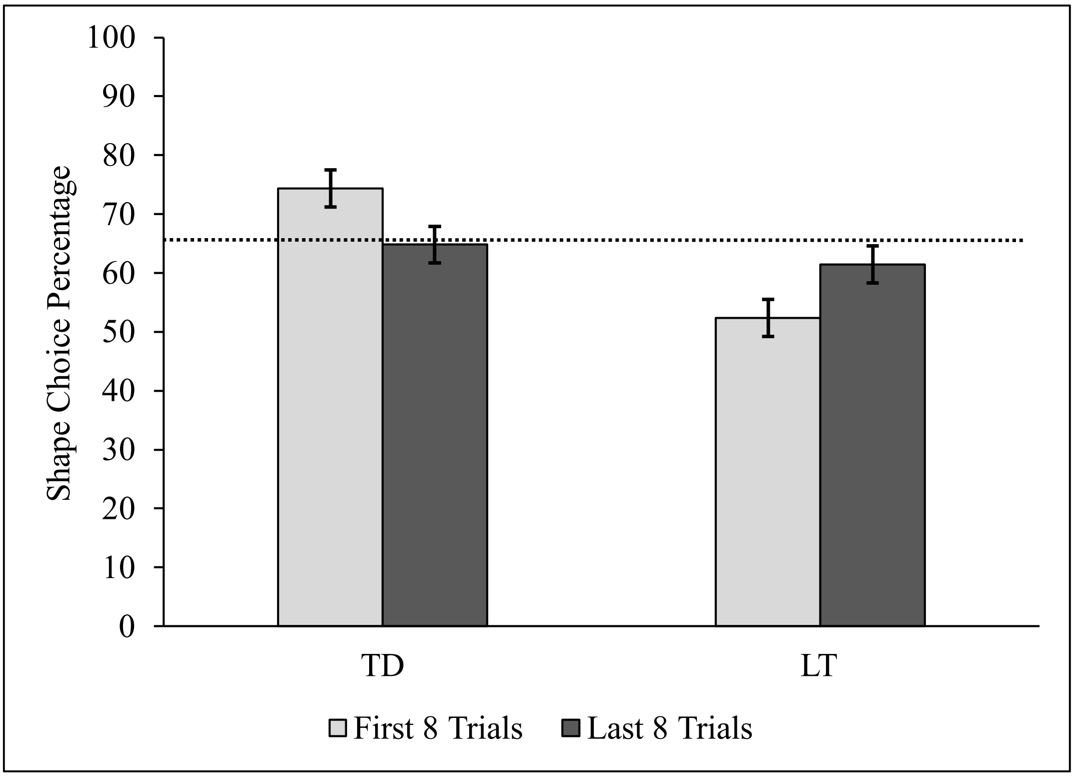

Note. The group-level shape-choice PCCs for typically developing (TD) and late talking (LT) children are reported in the bars for the first 8 and last 8 trials. The dashed line represents the median shape choice frequency (PCC_{mdn} = 65.48%) across all 64 children and all 16 trials. The error bars represent how much we could expect each PCC to deviate from physical chance.

Results for the LT infants show a different pattern. During the first 8 trials, the LT infants have a marginal preference to shape (PCC_{First8} = 52.34%, c = 0.24), which could plausibly be explained by physical chance. However, during the last 8 trials the LT infants show a stronger tendency to the shape-matched item (PCC_{Last8} = 61.44%, $c < .001$), that could not be plausibly explained by physical chance. Furthermore, the difference between the shape choice PCCs for LT infants' last 8 and first 8 trials (PCC_{diff} = 9.10%) could also not plausibly be explained by physical chance (c = .04). Given that the results demonstrated a trial effect between the TD and LT children, we further assessed whether the trial effects were apparent in our mixed-effects analysis between TD and LT infants separated by the three binned shape residual categories (below, nearly equal, and above expectations).

Table S2.
TD Children's Shape-Choice Results Separated by Trial Order

Case #	First 8 Trials			Last 8 Trials			All Trials Together		Shape Residual Category
	Trials	PCC	*c*-value	Trials	PCC	*c*-value	PCC	*c*-value	
1	8	50.00	0.633	8	62.50	0.363	56.25	0.40	B
2	8	75.00	0.142	8	62.50	0.360	68.75	0.10	B
3	7	71.43	0.147	5	60.00	0.365	66.67	0.10	B
4	7	42.86	0.642	8	37.50	0.853	40.00	0.78	B
5	5	80.00	0.036	7	71.43	0.140	75.00	0.04	A
6	7	85.71	0.032	7	57.14	0.371	71.43	0.04	NE
7	7	85.71	0.037	8	62.50	0.363	73.33	0.04	A
8	8	62.50	0.366	8	75.00	0.141	68.75	0.11	B
9	6	83.33	0.037	8	100.00	0.004	92.86	< 0.001	A
10	8	75.00	0.151	8	62.50	0.364	68.75	0.10	B
11	8	75.00	0.147	8	75.00	0.153	75.00	0.04	A
12	8	87.50	0.035	8	50.00	0.643	68.75	0.10	NE
13	8	75.00	0.145	8	75.00	0.140	75.00	0.04	B
14	8	75.00	0.146	8	50.00	0.641	62.50	0.24	A
15	4	50.00	0.636	4	75.00	0.149	62.50	0.23	A
16	4	100.00	0.005	2	100.00	0.005	100.0	< 0.001	NE
17	8	75.00	0.148	8	75.00	0.145	75.00	0.04	B
18	8	62.50	0.368	8	87.50	0.033	75.00	0.04	A
19	8	75.00	0.146	8	50.00	0.636	62.50	0.23	NE
20	8	62.50	0.363	8	75.00	0.138	68.75	0.10	NE
21	6	66.67	0.148	7	28.57	0.850	46.15	0.60	B
22	6	66.67	0.150	3	33.33	0.853	55.56	0.41	B
23	7	100.00	0.005	8	62.50	0.364	80.00	0.01	NE
24	8	75.00	0.144	8	37.50	0.859	56.25	0.40	NE
25	8	50.00	0.634	8	50.00	0.638	50.00	0.60	NE
26	8	100.00	0.004	8	87.50	0.037	93.75	< 0.001	NE
27	8	75.00	0.151	8	62.50	0.354	68.75	0.11	NE
28	8	75.00	0.149	6	66.67	0.141	71.43	0.04	NE
29	8	62.50	0.362	8	50.00	0.647	56.25	0.40	A
30	8	87.50	0.035	8	87.50	0.033	87.50	< 0.001	NE
31	8	100.00	0.004	8	62.50	0.371	81.25	0.01	NE
32	8	75.00	0.145	8	87.50	0.034	81.25	0.01	A
**Totals**	**234**	**74.35**	**< 0.001**	**233**	**64.81**	**< 0.001**	**69.59**	**< 0.001**	--

Note. Trial numbers vary because some children did not complete all sixteen trials. The chance-value (*c*-value) is from a randomization test with 10,000 iterations.
B = below expectations
NE = nearly equal to expectations
A = above expectations
PCC = Percent Correct Classifications index

Table S3.
LT Children's Shape-Choice Results Separated by Trial Order

Case #	First 8 Trials			Last 8 Trials			All Trials Together		Shape Residual Category
	Trials	PCC	*c*-value	Trials	PCC	*c*-value	PCC	*c*-value	
33	8	75	0.14	8	75	0.15	75.00	0.04	A
34	8	75	0.15	8	50	0.63	62.50	0.24	B
35	8	37.5	0.85	8	50	0.64	43.75*	0.78	B
36	5	40	0.63	6	33.333	0.86	36.36*	0.89	B
37	8	62.5	0.36	8	50	0.64	56.25	0.40	A
38	8	37.5	0.85	8	62.5	0.37	50.00*	0.60	A
39	7	28.571	0.86	6	66.667	0.15	46.15*	0.59	B
40	7	42.857	0.63	8	75	0.15	60.00	0.23	NE
41	7	28.571	0.85	8	37.5	0.86	33.33*	0.89	NE
42	8	50	0.65	4	75	0.15	58.33	0.23	A
43	8	37.5	0.85	7	85.714	0.04	60.00	0.23	NE
44	8	50	0.63	8	50	0.64	50.00*	0.60	NE
45	5	60	0.37	5	20	0.97	40.00*	0.78	A
46	8	100	0.005	8	87.5	0.03	93.75	< .0001	B
47	6	50	0.64	7	57.143	0.36	53.85	0.40	A
48	6	33.333	0.86	8	87.5	0.04	64.29	0.11	A
49	8	87.5	0.04	8	62.5	0.35	75.00	0.04	A
50	8	62.5	0.37	8	25	0.96	43.75*	0.77	B
51	8	75	0.14	6	66.667	0.14	71.43	0.04	A
52	8	50	0.64	8	50	0.65	50.00*	0.60	NE
53	6	66.667	0.15	0	.	1.00	66.67	0.11	B
54	5	40	0.64	6	66.667	0.15	54.55	0.40	NE
55	7	28.571	0.86	6	50	0.64	38.46*	0.77	A
56	8	0	1.00	8	62.5	0.36	31.25*	0.96	A
57	8	37.5	0.85	8	37.5	0.86	37.50*	0.90	B
58	6	66.667	0.15	3	100	0.004	77.78	0.01	**A**
59	8	75	0.15	8	75	0.14	75.00	0.04	NE
60	8	75	0.14	8	62.5	0.37	68.75	0.10	NE
61	8	50	0.64	8	50	0.64	50.00*	0.60	B
62	8	75	0.14	8	100	0.003	87.50	< .0001	NE
63	8	12.5	0.99	8	62.5	0.37	37.50*	0.90	B
64	8	50	0.63	7	85.714	0.03	66.67	0.11	B
**Totals**	**235**	**52.34**	**0.24**	**223**	**61.44**	**< 0.001**	**56.77**	**0.001**	**--**

Note. Trial numbers vary because some children did not complete all sixteen trials. The chance-value (*c*-value) is from a randomization test with 10,000 iterations.
B = below expectations
NE = nearly equal to expectations
A = above expectations
PCC = Percent Correct Classifications index

Re-evaluating the Mixed-Effects Analysis in Consideration of Trial Order Effects

In the original “mixed-effects” analysis presented in the main text, the overall patterns of PCCs for the TD and LT children did not demonstrate an interaction between the three shape residual conditions. When we separate by trial order (i.e., the first 8 and last 8 trials), however, there is visually compelling evidence of a 3-way interaction (i.e., a moderation effect) between the shape residual scores and talker status (see Figure S3 below). Specifically, during the first 8 trials the TD infants with shape residual scores below (PCC_{First8} = 66.22%, $c = .002$), nearly equal (PCC_{First8} = 81.63%, $c < .001$), and above (PCC_{First8} = 72.58%, $c < .001$) expectations chose the shape at higher frequencies.

During the last 8 trials, however, the TD infants with shape residuals below (PCC_{Last8} = 59.15%, $c = .04$) and nearly equal (PCC_{Last8} = 63.16%, $c = .003$) to expectations chose the shape at lower frequencies, whereas the TD infants with shape residuals above (PCC_{Last8} = 73.13%, $c < .001$) expectations chose shape at nearly the same frequency as the first 8 trials. Notably, the *c*-value computed for the difference from the NNG PCCs for the first 8 trials to the last 8 trials for the TD infants below expectations (PCC_{diff} = -7.07) was plausibly explainable by physical chance ($c = .39$). The TD infants whose shape residual scores were nearly equal to expectations, however, showed a larger change in their overall shape choice from the first 8 to the last 8 trials (PCC_{diff} = -18.47%) and this difference was not plausibly explainable by physical chance ($c = .01$).

Figure S3. Person-Centered Shape-Choice Results Split by Trial Order, Talker Status, and Shape Residual Categorization

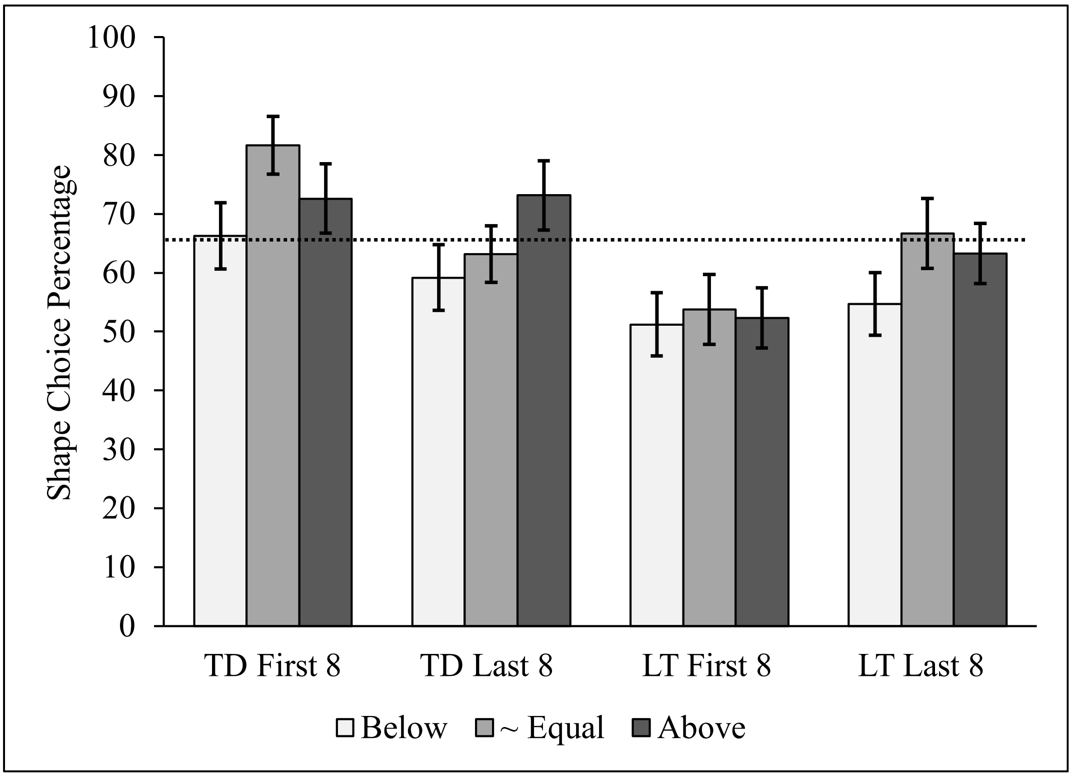

Note. The group-level shape-choice PCCs for typically developing (TD) and late talking (LT) children below, approximately equal, and above expected shape residual scores are reported in the bars for the first 8 and last 8 trials. The dashed line represents the median shape choice frequency (PCC_{mdn} = 65.48%) across all 64 children and all 16 trials. The error bars represent how much we could expect each PCC to deviate from physical chance.

The LT infants, however, showed a different pattern when compared to the TD infants. During the first 8 trials, the LT infants with shape residual scores nearly equal (PCC_{First8} = 53.73%, c = .28) and above (PCC_{First8} = 52.33%, c = .31) expectations chose the shape at frequencies only slightly above a 50% preference and could be plausibly explained by physical chance. During the last 8 trials, however, the LT infants with shape residuals nearly equal (PCC_{Last8} = 59.15%, c = .04) and above (PCC_{Last8} = 63.16%, c = .003) expectations chose the shape at higher frequencies that could not plausibly be explained by physical chance. Notably, the c-value computed for the difference from the NNG PCCs for the first 8 trials to the last 8 trials for the LT infants nearly equal to expectations (PCC_{diff} = 12.94%, c = .11) and above expectations (PCC_{diff} = 10.96, c = .15) were less plausibly explainable by physical chance. The LT infants with shape residuals below expectations chose shape at a similar frequency for the first 8 (PCC_{First8} = 51.22%, c = .38) and last 8

(PCC_{Last8} = 54.67%, c = 0.17) trials, which could be plausibly explained by physical chance in both instances.

These results therefore indicate stronger support for an interaction between the binned shape-based vocabulary and talking status, similar to those found initially by Perry and Kucker [4] prior to removing any outliers and only after considering the trial order effects. Of note, when one of the outliers (Case #46) was removed from our exploratory analyses the PCCs for the LT infants with shape residuals below expectations showed the same pattern, although the overall shape choices were lower from the first 8 (PCC_{First8} = 45.95) and last 8 (PCC_{Last8} = 50.75%) trials.

Summary of Exploratory Findings

In summary, there appears to be trial order effects present, specifically, during the first 8 Trials the TD infants, as a group, chose the shape item at higher rates than during the last 8 trials. The LT infants showed an opposite pattern, during the first 8 trials the LT infants, as a group, chose the shape item at a rate near 50%, but during the last 8 trials the LT infants shape-choice rates increased to above 60%. When we separated the TD and LT children by their shape-residual categories, even more information was obtained. The TD children with shape residual scores above expectations and the LT children with shape residual scores below expectations chose the shape item at nearly equal rates during the first 8 and last 8 trials, though the TD children chose the shape item at rates above 70% whereas the LT infants chose the shape item at rates near 50%. However, an interaction was observed such that the TD infants with shape residual scores below and nearly equal to expectations chose the shape-matched item at higher rates during the first 8 trials than during the last 8 trials. The LT infants also revealed an interaction effect, specifically, the LT infants with shape residuals nearly equal and above expectations initially chose the shape item at lower rates (~50%) during the first 8 trials but chose the shape item at rates higher than the median (< 65.48%) during the last 8 trials. Taken together, this suggests that behavioral responses from trial-to-trial matter (see also Samuelson & Horst [69]) and importantly, may shift in unique ways for LT vs. TD children. If one collapses

across all trials to get an overall performance score, we may see group differences, but this critical information is lost. Most importantly, this person-centered approach highlights a method for revealing relevant, but often neglected variables such as stimuli and trial order effects.
